# EARLY POSTOPERATIVE COMPLICATIONS IN ROUX-EN-Y GASTRIC BYPASS

**DOI:** 10.1590/0102-6720201600S10018

**Published:** 2016

**Authors:** Aluisio STOLL, Leandro ROSIN, Mariana Fernandes DIAS, Bruna MARQUIOTTI, Giovana GUGELMIN, Gabriela Fanezzi STOLL

**Affiliations:** UNIMED Hospital Center and Dona Helena Hospital, Joinville, SC, Brazil.

**Keywords:** Bariatric surgery, Postoperative complications, Gastric bypass

## Abstract

**Background:**

Roux-en-Y gastric bypass is one of the most common bariatric surgery and leads to considerable weight loss in the first months.

**Aim:**

To quantify the main early postoperative complications in patients submitted to the gastric bypass.

**Method:**

Observational retrospective cohort. Data of 1051 patients with class II obesity associated with comorbidities or class III obesity submitted to the gastric bypass with 30 days of follow-up starting from the date of the surgery.

**Results:**

The age average was 36 years with a predominance of females (81.1%). The mean preoperative body mass index was 43 kg/m². The major complication was fistula (2.3%), followed by intestinal obstruction (0.5%) and pulmonary embolism (0.5%). Death occurred in 0.6% of the cases.

**Conclusion:**

In the period of 30 days after surgery the overall complication rate was 3.8%; reoperation was necessary in 2.6% and death occurred in 0.6%. Fistula was the main complication and the leading cause of hospitalization in intensive care unit, reoperation and death.

## RESUMO

**Racional:**

Bypass gástrico em Y-de-Roux é uma das operações bariátricas mais comuns e leva a perdas consideráveis de peso já nos primeiros meses.

**Objetivo:**

Quantificar as principais complicações pós-operatórias precoces em pacientes submetidos ao bypass gástrico.

**Método:**

Coorte retrospectiva observacional. Amostra de 1051 pacientes portadores de obesidade grau II associada à comorbidades ou grau III submetidos ao bypass gástrico com acompanhamento de 30 dias a partir da data da operação.

**Resultados:**

A idade média dos pacientes foi de 36 anos com predominância de mulheres (81,1%). O índice de massa corporal pré-operatório médio foi de 43 kg/m². A principal complicação foi fístula (2,3%), seguida de obstrução intestinal (0,5%) e tromboembolismo pulmonar (0,5%). Óbito ocorreu em 0,6% dos casos.

**Conclusão:**

No período de 30 dias de pós-operatório a taxa geral de complicações foi de 3,8%; a de reoperação de 2,6% e óbito em 0,6%. A fístula foi a principal complicação e a principal causa de internamento em unidade de terapia intensiva, de reoperação e de óbito.

## INTRODUCTION

Obesity affects millions of people, thus being considered an epidemic^10^. In Brazil, around 40% of the population is overweight and around 10% of the total amount of public health budget is directed towards obese patients^10^. Obesity is characterized in patients whose body mass index (BMI) is >30 kg/m^2^. However, only patients with BMI ranging from 35-40 kg/m^2^ (class II obesity) with comorbidities or BMI>40 kg/m^2^ (class III obesity) are suitable candidates for bariatric surgery^10^ ^,^ ^11^.

Bariatric surgeries do not cure obesity, although they may contribute to excess weight reduction as well as improve certain comorbidities and mortality reduction because of weight excess^2^ ^,^ ^7^. Such procedures cause controlled undernutrition, which in turn tends to cause sustainable weight loss^3^ ^,^ ^4^.

One of the most common bariatric surgeries performed worldwide is Roux-en-Y gastric bypass (gastric bypass or gastric derivation), which is considered by many surgeons a gold standard procedure because of its safety and low complication rates^9^. This technique presents important antidiabetogenic effects with good results before the occurrence of great weight loss, which include an improvement in terms of glycemic control and the reduction of short-term use of hypoglycemiants^12^ ^,^ ^13^.

Regarding obesity operations, general complication rates vary from 10-17%, whereas reoperations correspond to an average of 7%, and death rate 0.008-0.35%^3^. In this context, despite gastric bypass' well-documented safety, there still are complications that may occur as result of the procedure^1^. 

Hence, this paper seeks to quantify the main early postoperative complications in patients submitted to gastric bypass, taking into consideration an early period of 30 days right after the operation.

## METHODS

The present study obtained an approval from Hans Dieter Schmidt's Regional Hospital Ethics Committee, registered under the number 47413415.4.0000.5363.

Observational retrospective cohort was adopted. The sample comprised 1051 patients with a BMI ranging from 35-40 kg/m² associated with comorbidities or IMC>40 kg/m² submitted to Roux-en-Y gastric bypass. Operations were performed at UNIMED Hospital Center and Dona Helena Hospital, both located in Joinville, SC, Brazil. Data were collected retrospectively. Analyses were carried out from November, 1999 until May, 2015, observing patients for 30 days after their operations. The variables analyzed were: gender, age upon surgery, weight and preoperative BMI, bariatric procedure conducted, and main early postoperative complications. The complications that were taken into account were: fistulas, intestinal obstructions, pulmonary thromboembolism, and factors that caused reoperations, hospitalization in the ICU or death.

All patients older than 16 years old with surgery indication and who were submitted to gastric bypass were included in the study. There was no exclusion criterion, since all of the patients met the criteria for valid surgery indication.

In the present study, continuous variables were described by means and standard deviations, whereas nominal and discrete variables by frequencies and percentages.

## RESULTS

Age range was 16-68 (36±10.3) years. Eight of them were 16-17 years old (16,6±0.5); 358 between 18-39 (30,4±5.3); 680 between 40-65 (48±6.1), and five were older than 65 (67,2±0.8). In the study, there was a total of 852 women (81.1%) and 199 men (18.9%). Preoperative BMI varied from 35-61.1 kg/m² (43±4.9) and preoperative weight varied from 80-198 kg (117.7±19).

In the study, with 236 patients with a BMI of 35-39.9 kg/m², systemic arterial hypertension was observed in 48.7% (n=115), dyslipidemia in 28% (n=66), diabetes mellitus - type 2 in 19.9% (n=47), obstructive sleep apnea in 11.4% (n=27), and gastroesophageal reflux disease in 5.9% (n=14).

Out of all of the 1051 patients, 40 of them (3.8%) presented complications. Among these, the main complication observed was fistula, which occurred in 24 of them (2.3%), followed by intestinal obstruction in five patients (0.5%) and thromboembolism in five patients as well (0.5%). Taking into account these 40 patients' BMI who presented complications, it was possible to perceive that most of them (n=30) had a BMI≥40 kg/m^2^, and complications were more common among these patients (Table 1).

**TABLE 1:** Complication rate in the first 30 postoperative days according to preoperative BMI

Complications	BMI from 35 to 40 kg/m^2^ (n=10)		BMI ≥40 kg/m^2^ (n=30)	
	Frequency	Percentage	Frequency	Percentage
Fistula	6	25%	18	75%
Intestinal obstruction	1	20%	4	80%
Thromboembolism	0	0%	5	100%
Reoperations	9	33%	18	67%
ICU	3	19%	13	81%
Death	1	17%	5	83%

**FIGURE 1:**
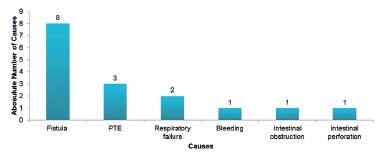
Causes of hospitalization in the ICU in the first 30 postoperative days

Hospitalization in the ICU was necessary for 16 patients (1.5%), eight of them due to fistula, three due to thromboembolism, two due to respiratory failure, one due to bleeding, one due to intestinal obstruction, and one due to intestinal perforation (Figure 1).

Reoperations were necessary in 27 patients (2.6%), 14 (51.9%) of which were due to fistula, five (18.5) due to intestinal obstruction, four (14.8%) due to bleeding, two (7.4%) due to suspicion of peritonitis, one (3.7%) due to intestinal perforation, and one (3.7%) due to necrohemorrhagic pancreatitis.

Death occurred in six (0.6%) patients, four of them due to fistula and two due to thromboembolism. Complication rate was analyzed from November, 1999 until May, 2016, and it was possible to determine that a major number of complications occurred in the first years, especially in 2001, 2002, 2003, and 2005 (Figure 3).

**FIGURE 2:**
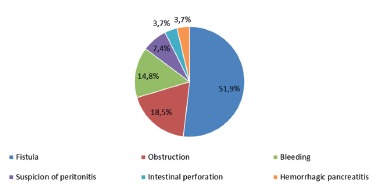
Causes of reoperation in the first 30 postoperative days

**FIGURE 3:**
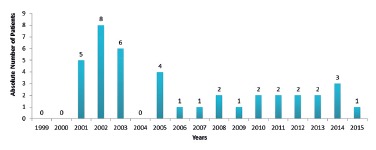
Complication rate in absolute number during the first 30 postoperative days from 1999 to 2015

## DISCUSSION

In the present research, 40 (3.8%) patients with some complication were found, 23 of which (2.2%) required reoperation. These numbers corroborate those obtained by the American College of Surgeons, in a publication containing an analysis of a period of 30 days regarding patients submitted to videolaparoscopic gastric bypass with complication rate higher than 3.3% and reoperation rate at 3.6%^5^.

The occurrence of fistula has been one of the most common early complications associated with gastric bypass, and the need to perform cholecystectomy as the main late complication^14^. The present study found that fistula was the most common complication, being present in 2.3% of the patients. 

Patients submitted to gastric bypass present both thromboembolism and deep venous thrombosis, around 2-4%^10^. In addition to obesity being a risk factor concerning thrombotic phenomena, BMI>40 kg/m² constitutes an independent risk factor for postoperative sudden death by thromboembolism ^10^. It was diagnosed in five patients (0.5%), all of which with BMI>40 kg/m^2^ and two of them died.

Mason et al.^6^, in an retrospective review of 38501 bariatric procedures published in 2007, found a mortality rate of 0.24% (93 deaths), with an emphasis on three main causes: thromboembolism (32%), fistula related complications (15%), and cardiac diseases (13%). The study showed that fistula was the leading cause of death, followed by thromboembolism.

Chang et al.^3^ found a mortality rate 30 days after gastric bypass procedures of 0.38% (0.22-0.59) (Chang). Similarly, death rate found was 0.6%.

Bariatric surgery is controversial with patients older than 65 years old. This is mainly due to evidence of higher surgical morbimortality in those patients^8^. In the present study, five older than 65 years old were in the sample, and only one of them presented complications, which was anastomosis of the fistula, followed by death.

## CONCLUSION

In the postoperative period of 30 days, the general complication rate was 3.8%; reoperation rate was 2.6%, and death rate was 0.6%. Fistula was the main complication observed as well as the main cause for hospitalization in the ICU, reoperation, and death.
